# The Peculiarities of Chronic Cocaine Poisoning

**Published:** 1889-04

**Authors:** Wm. C. Krauss

**Affiliations:** Attica, N. Y.; 5 Rue Rollin, Paris


### *THE PECULIARITIES OF CHRONIC COCAINE POISONING.*

Reported by Dr. WM. C. KRAUSS, of Attica, N. Y.

In a lecture before his class, at the Asile St. Anne, Paris, M. Magnan reported the results of his observations on chronic cocaine poisoning (the same observations were communicated to the *Société de Biologie* under the presidency of Brown-Sequard, January 26, 1889).

Professor Magnan had three cases under observation:

First Case: A merchant, forty-eight years old, began using morphine in 1878 for nephritic colics. In 1886, to rid himself of the morphine habit, he resorted to the chlorhydrate of cocaine, and in a short space of time was taking heavy doses. Frightened by the peculiar sensations produced by the cocaine, he abandoned it after a lapse of two months. His sensations were illusory; objects seemed to move about him; he complained of auditory hallucinations, hearing suddenly strange sounds, which frightened him, etc. The hyper-excitability, neuro-muscular, revealed itself by spasms of the extremities. The patient reverted to his injections of morphine, but at the end of six months abandoned them for the cocaine, and, as a consequence, the hallucinations returned. He imagines having a foreign body under the skin, feels somebody striking him, etc. An examination of the general sensibility shows it to be somewhat analgetic. At the end of several weeks, epileptic attacks set in, from which patient is still suffering.

Observation 2: A pharmaceutist, forty-four years old, began the use of morphine in 1884 for hepatic colics. In 1887, he tried cocaine. Hallucinations of the general sensibility appeared; he believes his skin infected with microbes, shows muscular spasms, tremor; suddenly an epileptic attack occurs, which is followed soon after by recurrent attacks.

Observation 3: A physician, thirty-nine years old, began the injections of morphine in 1872 for a severe neuralgia. During this time he has never experienced any sensorial troubles. In 1887 he began the use of cocaine, gradually increasing the dose. After a short time he complained of hallucinations of sight and of audition, sensation of strange bodies under the skin, slight analgesia, etc.

From these three observations Professor Magnan concludes that cocaine produces most frequently troubles of the general sensibility, followed by hallucinations. The action of cocaine is, therefore, very different from that of morphine, and resembles rather the alcohols and absinthe. From the latter it differs, however, in that the intoxication from cocaine acting upon the cortex of the brain, appears to progress from behind forwards, beginning at the occipital lobe and gradually invading the sensorial centers. The reverse of this occurs in alcohol and absinthe intoxication.

5 Rue Rollin, Paris.

